# Daily Eating Frequency in US Adults: Associations with Low-Calorie Sweeteners, Body Mass Index, and Nutrient Intake (NHANES 2007–2016)

**DOI:** 10.3390/nu12092566

**Published:** 2020-08-24

**Authors:** Kelly J. Hunt, John V. St. Peter, Angela M. Malek, Caroline Vrana-Diaz, Bernadette P. Marriott, Danielle Greenberg

**Affiliations:** 1 Department of Public Health Sciences, Medical University of South Carolina, Charleston, SC 29425, USA; huntke@musc.edu (K.J.H.); malek@musc.edu (A.M.M.); vrana@musc.edu (C.V.-D.); 2 PepsiCo R&D, PepsiCo, Inc., Purchase, NY 10577, USA; stpet003@umn.edu; 3 Department of Experimental & Clinical Pharmacology, College of Pharmacy, University of Minnesota, Minneapolis, MN 55455, USA; 4 Departments of Medicine and Psychiatry, Medical University of South Carolina, Charleston, SC 29425, USA; marriobp@musc.edu; 5 NutriSci Inc., Mt. Kisco, NY 10549, USA

**Keywords:** National Health and Nutrition Examination Survey (NHANES), eating frequency, low-calorie sweeteners, artificial sweeteners, nutritive sweeteners, body mass index (BMI), eating episodes

## Abstract

Studies of relationships between eating frequency and/or timing and energy intake have not examined associations with low-calorie sweeteners (LCS). We assessed the frequency of eating behavior related to LCS consumption emphasizing timing, calorie intake, and body mass index (BMI) among United States (US) adults aged ≥19 years. Using the National Health and Nutrition Examination Survey (NHANES) 2007–2016, we defined eating episodes as food and/or beverage intake within 15 min of one another over the first 24-h dietary recall. We coded items ingested during episodes (*n* = 136,938) and assessed LCS presence using US Department of Agriculture (USDA) food files. Episode analysis found intakes of foods only (27.4%), beverages only (29.5%), and foods with beverages (43.0%). LCS items were consumed without concurrent calories from other sources in fewer than 2.7% of all episodes. Within participants having normal weight (29.4%), overweight (33.6%) and obese (37.1%) BMIs, LCS consumers (35.2% overall) evidenced: more episodes/day; and fewer: calories, carbohydrates, fats, and protein per episode. Per person, those consuming LCS had lower total calories and higher fiber intake per day. LCS consumption was associated with higher BMI. Number of eating episodes/day and longer hours when eating episodes occurred were also consistently associated with higher BMI. Consuming LCS did not modify these relationships. These results did not show that LCS consumption was associated with increased caloric intake from other dietary sources.

## 1. Introduction 

One of the main topics of the 2020 Dietary Guidelines Advisory Committee (DGAC) is a focus on “frequency of eating”. Specifically, they ask: “What is the relationship between the frequency of eating (such as meals per day, snacking, and fasting) at each stage of life and achieving nutrient and food group recommendations?” [1]. This study aims to describe how this 2020 DGAC question might be addressed using the National Health and Nutrition Examination Survey (NHANES) data with a specific emphasis on how frequency of eating low-calorie sweeteners (LCS) is exhibited in the diet.

At the national level, in the United States (US), the Centers for Disease Control and Prevention (CDC) is continually conducting the NHANES which includes two 24-h dietary recalls [2]. These data form an important source of information about food and nutrient intake by the US population and the relation to health outcomes. An additional application for the NHANES 24-h recall dietary data is to use the time stamp within the data collection to understand patterns and frequency of eating in the US population [3,4,5]. Previous studies have examined various eating patterns using NHANES for the association between eating occasions, eating frequency and body mass index (BMI), sleep duration, and nutrient profile [6,7,8,9,10,11,12]. However, no studies to date have considered eating frequency patterns and consumption of LCS along with possible association with BMI. While the definitions of an eating occasion are variable [13], over the past three decades, estimated daily eating frequency [14] as well as estimated total intake of LCS containing foods have been an area of much interest. 

Eating frequency has been associated with energy intake in previous studies [6,15,16]. Some studies have reported that LCS consumption is increasing dramatically [17,18]. However, while incorporation of LCS into the number of food and beverage items may be increasing, more direct measures of the quantity of human consumption seem to indicate stable or declining ingestion. For example, sales data show that diet soft drink consumption, which accounts for about 51% of total LCS usage globally, has been declining steeply [19]. In addition, available US Department of Agriculture (USDA) data suggest the total available tonnage of LCS is increasing but at relatively moderate rates [20,21]. LCS-containing foods and beverages, in addition to providing fewer overall calories than those containing nutritive sweeteners (NS) including all types of added sugars, have been associated with healthier lifestyle choices, such as increased physical activity, greater fruit and vegetable intake, reduced energy intake, and higher Healthy Eating Index scores [22,23]. LCS have also been used as tools for weight loss, weight loss maintenance, and diabetes management [24,25,26,27,28].

The relationship between BMI and LCS consumption is inconsistent, as some have reported an inverse association [18,29,30,31], while others have not observed this finding [24,27,32]. The role of LCS in weight management remains controversial as randomized controlled trials typically find LCS to be of benefit in weight loss programs [24,27,32], yet some cohort studies associate LCS consumption with higher body weight [30,31,33]. Studies in animals also show conflicting results of the relationship between LCS consumption and body weight [24]. In their meta-analysis of animal studies, Rogers et al. note, “Evidence from a large majority of animal studies favors reduced energy intake and body weight gain or no effect of low energy sweeteners, although increases have been observed as well” [24,34,35,36,37]. Conflicting findings are not surprising, as experimental paradigms used in animal investigations may not accurately reflect actual human LCS consumption patterns, and the paradigms are not consistent across studies. Clarity will require further study of the relationship between human eating frequency, body weight, and LCS intake. 

The aim of our analysis was to use time-specific records in the nationally representative NHANES dietary data to assess eating frequency and consumption of LCS by US adults based on the 24-h intake recall measure in NHANES. Others have investigated the effects of small frequent meals compared to three larger meals on body weight. A meta-analysis of these studies suggests, however, that this is still an open question [38]. We examined behavior with respect to characteristics of individual eating occasions termed eating episodes, as well as the total number of eating episodes, ratio of evening to morning energy intake, and total number of hours during which eating episodes were reported. Specifically, we examined macronutrient and calorie intake by presence or absence of LCS within eating episodes and examined relationships to eating frequencies within the BMI category. We hypothesized that individuals who consumed LCS containing foods, beverages or food and beverage additions (FBAs) within eating episodes may have different eating frequencies than those who did not, either alone or in relationship to BMI. 

## 2. Subjects and Methods

We used the first 24-h, interviewer-based, dietary recall from five consecutive cycles of the NHANES, 2007–2016. To ensure representativeness of the US population, the National Center for Health Statistics (NCHS) designed a complex, multistage probability sample for each NHANES continuous cycle (2007–2008, 2009–2010, 2011–2012, 2013–2014, 2015–2016). The NHANES cycles (2007–2016) in this analysis included a total of 25,411 US adults (49.6% males, *n* = 12,733; 50.4% females, *n* = 12,678) aged ≥19 years (y) (weighted N = 217,802,638) with normal weight, overweight or obese BMIs. Pregnant and lactating females, individuals classed as underweight BMI, and those with missing or incomplete dietary records were excluded (*n* = 4556) (see Supplementary Figure S1). This NHANES data analysis was not considered human subjects research by the Medical University of South Carolina Institutional Review Board for Human Research.

### 2.1. Definitions

We classified race–ethnicity as: non-Hispanic white (NHW), non-Hispanic black (NHB), Hispanic or Other, including Non-Hispanic Asians and persons of other non-Hispanic race or non-Hispanic multi-race [39]; age as: 19–30, 31–50, 51–70, and 71–80 y; BMI (kg/m^2^) as: underweight (<18.5) normal weight (18.5–24.9), overweight (25–29.9) or obese (≥30); and educational level as: less than high school graduate or passing the General Educational Development Test, some college or Associates Degree or college graduate or higher. We assessed socioeconomic status using the poverty income ratio, where income is divided by the threshold, and categorized as: below the poverty line, at or above the poverty line or 3 times above the poverty line.

Self-reported ingested items were recorded for each cycle (*n* = 402,930 items; 87,270 from 2007 to 2008, 91,460 from 2009 to 2010, 72,466 from 2011 to 2012, 78,729 from 2013 to 2014, and 73,005 from 2015 to 2016) by trained NHANES dietary interviewers. Items were coded as food, beverage, and/or FBAs and categorized as including LCS and/or NS as previously described and outlined below [40]. FBAs were defined as food or beverage items that were added to or consumed with foods or beverages rather than consumed alone (e.g., syrup, table top sweeteners, mustard, salad dressing) [41]. Foods, beverages, and FBA items were categorized as containing LCS according to the USDA item descriptions of “with low/no calorie sweetener”, “sugar-free” or “dietetic/low sugar” and/or as containing NS if caloric sweetener was present. Caloric sweeteners included all items noted in the US Food and Drug Administration (FDA) guidance on added sugars [42]. If the presence of LCS in a food, beverage or FBA item was unclear, it was verified through internet food label searches [40]. Similar to earlier studies [16,43], this study used the first 24-h dietary recall from NHANES. Interviewee-defined eating occasions were used in the analysis of eating episodes based on the times that the respondent indicated the start of an “eating episode”; episode ending times were not collected by NHANES. An eating episode was defined as food and/or beverage items consumed within 15 min of one another over the 24-h recall [14]. Eating episodes were classified as food only, beverage only or foods and beverages together. Eating episodes could contain NS and LCS inherent in foods or beverages as well as NS and LCS that were added as FBAs. Included in our analysis were the reported number of overall eating episodes as well as those with food alone, beverages alone, and foods and beverages together. Of the 137,023 eating episodes, 85 were excluded because no food or beverage was consumed (i.e., an FBA was consumed alone, such as a small amount of peanut butter, icing or cheese spread) for a total of 136,938 eating episodes (weighted N = 1,219,061,667) included in our analysis.

We examined eating frequency and consumption patterns using several approaches. First, we examined eating episode characteristics including their content and chronology. With respect to eating episode content, we examined: (1) whether the episode comprised food, beverage or a combination thereof; (2) whether LCS and/or NS was included; and (3) the nutrient contents of the episode. Consumption of LCS and NS within eating episodes was dichotomized as yes or no over the 24-h period and within a specific episode. With respect to nutrient content, we combined the data in the dietary interview component of the NHANES *What We Eat in America* with the relevant items in the USDA’s Food and Nutrient Database for Dietary Studies. This database provides gram amounts to enable conversion of NHANES food and beverage intake information into estimated nutrient values [44,45,46]. With respect to chronology, at the person level, we examined the average number of eating episodes per day as well as timing of the first and last eating episode. We also examined hours during which eating episodes occurred per day defined as 24 h minus the largest gap between eating episodes. Hence, if the largest gap between eating episodes occurred between 10:00 p.m. and 6:00 a.m. (i.e., 8 h), the number of hours during which eating episodes occurred would be 16 h. Individuals were categorized into four groups based on hours eating per day using quartile cut-off points: less than 11.0 h per 24 h, ≥11.0 to <13.0 h per 24 h, ≥13.0 to <14.5 h per 24 h or 14.5 h or greater per 24 h. We additionally examined evening/morning energy ratio that was defined as energy intake (kcal) between 6:00 and 11:55 p.m. for evening intake divided by energy intake (kcal) between 6:00 and 11:55 a.m. for morning intake plus one, according to Aljuraiban et al.’s definition [9]. Participants were categorized into four groups based on their evening/morning energy ratio using quartiles (i.e., <0.73, ≥0.73 to <1.69, ≥1.69 to <4.00, and ≥4.00).

### 2.2. Statistical Analysis

Descriptive statistics, including Wald chi-square tests for categorical data and t-tests to compare mean differences for continuous variables, were performed to compare eating episodes by LCS consumption, and by LCS consumption stratified by BMI category. Demographic factors were examined in the total population and stratified by LCS. Analyses were performed from two different perspectives: eating episode and person level. Eating frequency variables were examined in the total population and stratified by LCS consumption and BMI category. Dietary intake levels of nutrients (g), energy distribution (kcal), and energy density (kcal/g) were also examined by eating episode and across all eating episodes stratified at the person level by LCS consumption and BMI category. All analyses accounted for oversampling and the clustered sampling design, and use of five continuous NHANES cycles adjusted for non-response and differential non-coverage [47]. The USDA *What We Eat in America* food files, which contain sample weights, were used to evaluate food, beverage, and FBA items consumed in self-reported eating episodes [48]. 

Separate linear regression models were used to examine mean BMI differences (i.e., dependent variable) in relationship to each eating behavior variable (i.e., LCS consumption, number of eating episodes per day, hours during which eating episodes occurred, and evening/morning energy ratio). Models were initially adjusted for demographic factors (i.e., age category, gender, race–ethnic group and education level) with subsequent models being additionally adjusted for LCS as well as total energy consumption. Moreover, to determine whether LCS consumption frequency modified the association between each of the other eating behavior variables, appropriate interaction terms were used. All analyses were conducted using SAS, version 9.4 and its complex survey-specific procedures (SAS institute; Cary, NC, USA). 

## 3. Results

**Figure 1:**
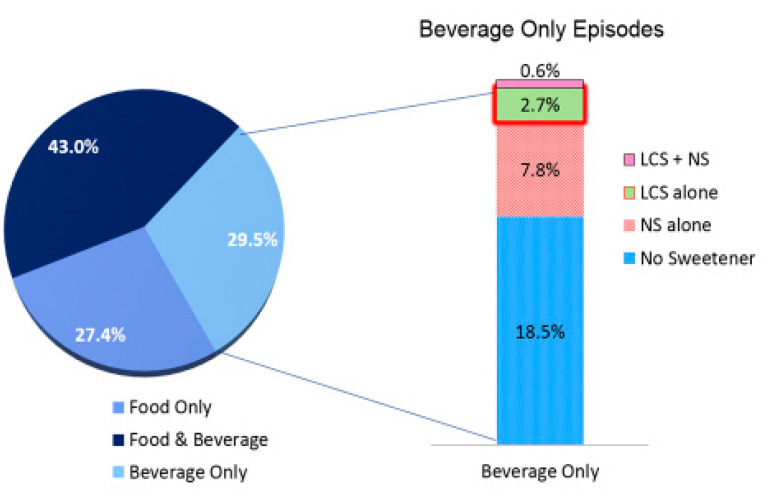
Prevalence of low-calorie sweetener (LCS) contained in eating episodes by type of eating episode and comparison of beverage only eating episodes by LCS and nutritive sweetener (NS) status (NHANES 2007–2016; eating episode *n* = 136,938; weighted N = 1,219,061,667).

Sample demographics are summarized in Supplementary Table S1. The unweighted 1-day sample included 25,411 individuals who consumed a total of 402,930 food, beverage or FBA items during 136,938 eating episodes. The largest number of eating episodes reported by an individual within a 24-h period was 18. Overall, 35.2% of participants consumed foods, beverages or FBAs with LCS present. Individuals who consumed LCS were more likely to be female, NHW, age 51 y or older, a college graduate, obese, and have a higher income (i.e., 3× above poverty line) than individuals who did not consume LCS (*p* < 0.001). Characterizing eating episode content, Figure 1 depicts the food versus beverage composition of eating episodes and prevalence of LCS and NS containing items. This analysis was performed for eating episodes comprising food only, beverages only, and food and beverages together. Food only eating episodes comprised 27.4% of all episodes, beverage only eating episodes comprised 29.5% of all episodes, and food combined with beverage eating episodes comprised 43.0% of all eating episodes. Within the eating episodes where beverages only were consumed (29.5%), those episodes where beverages contained no sweetener were predominant (18.5% of all episodes; 62.7% of beverage only episodes). Beverages containing NS but no LCS were less frequent (7.8% of all episodes; 26.4% of beverage only episodes). Beverages containing LCS but no NS were less common (2.7% of all episodes; 9.2% of beverage only episodes). Episodes where beverages contained both NS and LCS were rare (0.6% of all episodes; 2.0% of beverage only episodes). Significantly, only 2.7% of all eating episodes occurred where LCS was reported as consumed without concurrent calories from other sources (i.e., beverage only eating episodes with beverages containing LCS but not NS or other calorie containing foods). We found that 11.3% of all eating episodes contained some item (either a food, a beverage or a combination) that contained LCS; of these, 23.6% occurred where LCS was reported as consumed without concurrent calories. 

Focusing on LCS and NS containing foods, beverages or FBAs reported within episodes, 53.1% of all eating episodes include NS only, 4.9% include LCS only, 6.4% include LCS and NS, and 35.7% include neither LCS nor NS (Table 1). The average number of eating episodes consumed per day was 5.6 ± 0.03, with eating episode consumption starting on average at 7:30 a.m., ending on average at 8:23 p.m., and time between first and last eating episode being an average of 12.6 ± 0.04 h (Table 1).

In those who did not consume LCS, a higher proportion of eating episodes were free of sweetener containing items (i.e., LCS and/or NS) compared to those who consumed LCS. This was true for all BMI categories: normal weight, overweight, and obese individuals (Table 1). In LCS non-consumers, in normal weight, overweight, and obese individuals, 38.8%, 38.4%, and 38.7% of eating episodes were free of sweeteners (i.e., either LCS or NS), respectively. In contrast, LCS consumers had a lower percentage of eating episodes without NS or LCS across all BMI categories (i.e., 30.4% in normal weight; 31.0% in overweight; and 30.5% in obese). 

Those reporting LCS consumption had more eating episodes during more hours of the day than those who did not consume LCS, regardless of BMI category (*p* < 0.001) (see Table 1). Within each BMI category, the time of the first eating episode was significantly earlier in those consuming LCS than those not consuming LCS in overweight and obese individuals; and the time of the last eating episode was later in LCS consumers than non-consumers in obese, but not normal weight or overweight individuals. Within each BMI category, the total number of hours during which eating episodes occurred were greater in those who consumed LCS compared to those not consuming LCS. The average evening/morning energy ratio, with low values indicating more ingestion in the morning, was lower in normal and overweight individuals who consumed LCS than in non-consumers, but higher in obese individuals who consumed LCS than in non-consumers. 

**Figure 2:**
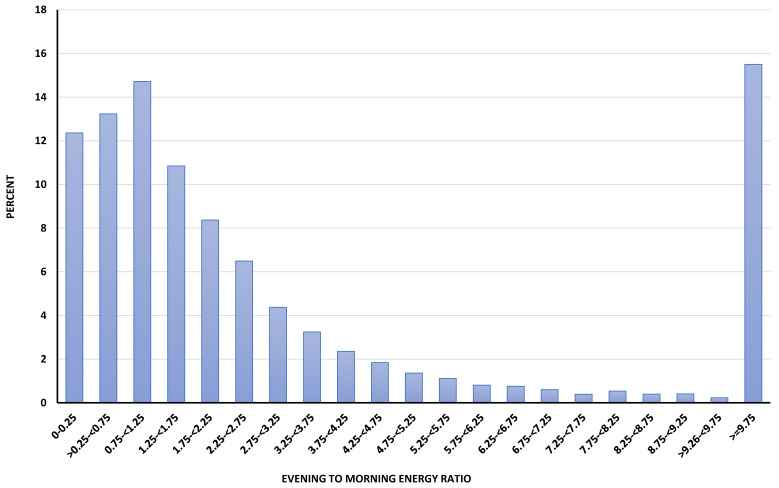
Histogram of evening to morning energy ratios. Evening/morning energy ratio was defined as energy intake between 6:00 and 11:55 p.m. (kcal) for evening intake divided by energy intake between 6:00 and 11:55 a.m. (kcal) for morning intake plus one according to Aljuraiban et al.’s definition [9]. Participants were categorized into four groups based on their evening/morning energy ratio using quartiles as cut-off points (<0.73; ≥0.73 to <1.69; ≥1.69 to <4.00; and ≥4.00). Individuals with lower values tended to consume more calories in the morning than evening, while individuals with higher values tended to consume more calories in the evening than morning (NHANES 2007–2016; *n* = 25,411; weighted N = 217,802,638).

Evening/morning energy ratio ranged from 0 to 7409, with low values indicating more ingestion in the morning and high values indicating more ingestion in the evening (Figure 2). With quartiles used as cut-off points for the categorical variables, evening energy intake was between 0 and 0.73 times that of morning energy intake for the first quartile and the evening energy intake was at least 4.00 times that of morning energy intake in the fourth quartile.

Nutrients (g) and energy distribution (kcal) compared by LCS and BMI category are shown in Table 2. Differences in nutrient intake were observed at the eating episode level between LCS and non-LCS consumers for total grams consumed, energy, protein, carbohydrate, total sugars, and total fat, regardless of BMI category (all *p* < 0.05). Energy from food and beverages was lower per eating episode in LCS consumers compared to non-consumers regardless of BMI category (*p* < 0.001); however, energy from FBAs did not differ by LCS consumption. Differences in nutrient intake were observed for all eating episodes across the day (i.e., at the person level), except for protein, which did not differ by LCS consumption in overweight individuals. Total fat did not differ by LCS consumption in obese persons, and dietary fiber was higher in those who consumed LCS than in non-consumers. Of note, total energy intake for LCS consumers was 1977 ± 26 kcal for those with normal weight, 2075 ± 26 for overweight, and 2055 ± 20 for obese individuals compared with non-LCS consumers (2225 ± 24, 2220 ± 19, and 2147 ± 19, respectively; *p* < 0.001). 

Results of the multivariate models estimating mean BMI differences per indicated unit of eating behavior variables following adjustment are displayed in Table 3. LCS consumers had a BMI 1.61 (95% confidence interval (CI): 1.32, 1.91) kg/m^2^ greater than non-consumers after adjustment for age category, gender, and race–ethnic group. Each additional eating episode reported by a participant was associated with a decrement in BMI (0.16 (95% CI: 0.23, 0.08) kg/m^2^). Moreover, this relationship held after adjusting for LCS consumption (−0.20 (95% CI: −0.27, −0.12)) as well as total energy consumption (−0.22 (95% CI: −0.29, −0.14)).

The continuous variable for hours during which eating episodes were reported indicated that for each additional hour, there was an associated BMI decrease of 0.05 (95% CI: −0.10, −0.001) kg/m^2^ after adjustment for LCS and total energy consumption. Categorical results for hours during which eating episodes were reported reflect the same pattern, namely that increased hours were associated with decreased BMI after adjustment for LCS and total energy consumption. Focusing on the categorical variable for evening/morning energy ratio, comparing the third to first quartile, those in the third quartile (i.e., ratio from 1.69 to 4.00) had a BMI 0.42 (95% CI: 0.10, 0.74) kg/m^2^ lower in the final model.

Interaction terms were used to examine whether LCS consumption modified the association between model variables and BMI (Table 4). While each additional eating episode remained associated with a decrease in BMI level in either group, there was no evidence that LCS consumption modified this association. LCS consumption also did not modify the relationship between evening/morning energy ratio and BMI. Additionally, we found no consistent evidence that LCS consumption modified the association between hours during which eating episodes occurred and BMI. Results indicated no evidence of interaction when hours during which eating occurred were modeled as a continuous variable (*p* = 0.4231), but there was potential evidence of an interaction when these hours were modeled as a categorical variable (global *p*-value across the four categories = 0.0248), with the 11 to 13 h eating category being the only category indicating potential evidence of interaction (*p* = 0.0035). 

## 4. Discussion

Individuals who reported intake of food, beverage, and FBA items containing LCS had significantly more eating episodes per day and a greater number of total daily hours during which eating episodes were reported, regardless of BMI category. Overweight and obese individuals consuming LCS also had an earlier first eating episode in the morning than non-consumers. Analysis at the person level showed differences in macronutrient intakes as those consuming LCS ate fewer carbohydrates along with more fiber. Calorie intake was lower per eating episode for LCS consumers. Calorie intake was also lower across all eating episodes for the day, resulting in an association with lower total calorie intake in LCS consumers. Intake of calories, carbohydrates, and total sugars were lower in LCS consumers for all BMI categories. In overweight individuals, the distribution of evening/morning energy ratio also differed by LCS consumption. 

An important observation from our assessment of human LCS item consumption is that only 2.7% of all eating episodes identified LCS item consumption without the presence of calories and the resulting post-ingestive consequences. Concerns about weight gain associated with LCS consumption are sometimes linked to widely cited experimental animal study paradigms. These studies suggest Pavlovian conditioning will result from sweet tastes being associated with a lack of post-ingestive caloric consequences when LCS are present [34,35,36]. This possible conditioning is evidenced by excess consumption and consequent weight gain in rodents [34,35,36]. A necessary requisite for Pavlovian conditioning is consistent pairing of the conditioning stimulus (i.e., sweet taste) with the unconditioned response [49]. This consistent pairing with LCS present is a hallmark of the rodent studies. Our findings in human eating behavior observed from the NHANES datasets suggest that the requisite consistency of LCS consumption paired with absent calories is not paralleled. As shown in Figure 2, the only situation wherein LCS are consumed with no caloric consequences is in the beverage alone with LCS situation, which represented fewer than 3% of eating episodes. Uncoupling of sweet taste from post-ingestive consequences of calories, in rodent studies, leads to a weakening of conditioned responses to sweet tastes [50]. The possibility of weight gain in response to this uncoupling hypothesis states that disassociating sweet taste from energy results in an impaired ability to use sweet taste to control food intake [50]. A recent human study failed to substantiate this uncoupling hypothesis [51]. The rarity of LCS being paired with no post-ingestive consequences from other caloric sources suggests that human behaviors may differ from those observed in the animal studies demonstrating weight gain with LCS consumption. 

Few studies have investigated the relationship between the ratio of evening/morning energy intake and BMI [6,7,9], and none have examined the association in relation to consumption of LCS containing items. A relationship was observed for mean BMI differences and the ratio of evening/morning energy intake (0.2; 95% CI: 0.04, 0.2) in the International Study on Macro/Micronutrients and Blood Pressure (INTERMAP) after adjustment for sex, age, and population sample [9]. Intake of large morning versus evening meals and changes in body weight were also examined in a US study involving ten obese women [52]. These authors reported greater weight loss with larger morning meals, while intake of larger evening meals was important in preserving fat-free mass [52]. An association for percent energy from evening food intake and BMI was not found after adjustment for covariates in another US study of 1802 women aged 19–50, based on the USDA Continuing Survey of Food Intakes of Individuals (CSFII), in which evening food intake was related to percentage of energy from grams of alcohol, fat, and protein [3]. Additionally, when compared to day workers, day–night rotation workers have been reported to have a higher prevalence of obesity and overweight as well as higher BMIs [53,54]. These individuals also have increased evening food intake [55]. 

Our investigation found an association between evening/morning energy ratio and lower BMI regardless of consumption of LCS. We did not find evidence that reported consumption of LCS containing foods or beverages modified the relationship between any of the eating behaviors or timing examined (i.e., eating frequency, hours during which eating episodes occurred or evening/morning energy ratio) and BMI. Hence, the relationship between examined consumption patterns and BMI appears to be unaffected by the choice to include or not include LCS containing foods or beverages. It should be noted that in the current manuscript, we have focused mainly on the population level of LCS consumption eating episodes rather than the person level. The distribution at the person level was discussed in previous work which examined the distribution of LCS use within LCS consumers [5,41].

Obesity has been shown to be influenced by eating frequency [56]. Conflicting results have been reported in previous studies that investigated BMI and daytime eating frequency, with some showing increased daily intake and lower BMI [9,10,56,57] or adiposity index [58], whereas others showed increased BMI [59] or failed to find associations [4,60,61]. However, replication attempts also have been inconsistent [11,59]. Our results are consistent with those of previous studies reporting an association between increased number of eating episodes and decreased BMI [6,9,10], in that we found BMI decreased by 0.16 units for each additional eating episode reported, and no differences were observed with regard to consumption of LCS. 

Using data from sources other than NHANES, previous researchers have investigated time spent eating as a primary or secondary activity [62]. Total eating time (primary and secondary) was found to increase for both men (2.0 to 2.4 h/d) and women (1.6 and 2.5 h/d) from 1975 to 2006–2007, during which the risk for overweight and obesity also increased [62]. In the absence of directly measured eating duration, our analysis examined total hours during which eating episodes were reported by BMI and LCS item consumption. We similarly found an association with BMI after adjustment for covariates and consuming LCS items did not modify this relationship. 

Although our study has strengths due to the large sample size that is representative of the US population from 2007 through 2016 [63], these same data are known to have limitations [64] that may result in our estimates of the intake of macronutrients and LCS/NS items being underestimated due to underreporting [44,65]. We attempted to reduce misclassification as individuals were grouped according to reported intake of LCS in food, beverage, and FBAs. We also acknowledge that intake of LCS or market availability in food, beverage, and FBA items may differ as the current study involved dietary data from five continuous cycles of NHANES (2007–2016) and the most current version of USDA’s Food and Nutrient Database for Dietary Studies, 5.0 [64]. Finally, the cross-sectional study design limits the scope to associations and prevents examination of causal relationships between factors. 

## 5. Conclusions

Data from these five nationally representative NHANES cycles in US adults showed that the number of eating episodes per day, as well as the evening/morning energy ratio, were associated with BMI, as was time during which eating episodes were reported in a 24-h period. Frequency and timing of eating consistently differed by consumption status of LCS. We found, however, no evidence that consuming LCS containing items modified the relationship between any of the various eating behaviors examined and BMI. We found that human consumers of LCS demonstrated overall lower caloric and carbohydrate intake compared to non-consumers, contrasting findings from animal studies. Examination of eating episodes using the NHANES 24-h recall data not only appears to be a fruitful source of information regarding consumption of LCS but also offers potential for increased insight regarding the eating frequency patterns in US adults in general.

Data described in the manuscript, will be made available upon request pending application and approval.

## Figures and Tables

**Table 1:** Eating episode level *^1^* and person level *^2^* ingestion characteristics (i.e., mean or percent ± standard error; median (25th, 75th percentile) across the day in US adults aged ≥19 y by LCS intake and BMI category (NHANES 2007–2016). *^3.^*

	Total Population	Total Population		Normal Weight (29.4%)		Overweight (33.6%)		Obese (37.1%)	
		No LCS (64.8%)	LCS (35.2%)	No LCS (21.2%)	LCS (8.2%)	No LCS (21.7%)	LCS (11.9%)	No LCS (21.9%)	LCS (15.1%)
Type of Episode *^4^* (%)									
Food Only	27.44 ± 0.40	27.91 ± 0.42	26.65 ± 0.50 *	29.06 ± 0.52	27.43 ± 0.92 *	27.47 ± 0.46	26.09 ± 0.74	27.19 ± 0.60	26.66 ± 0.64
Food and Beverage	43.01 ± 0.38	42.48 ± 0.43	43.90 ± 0.46	41.23 ± 0.53	43.84 ± 0.75	43.14 ± 0.53	44.42 ± 0.63	43.09 ± 0.56	43.52 ± 0.67
Beverage Only	29.55 ± 0.30	29.61 ± 0.30	29.45 ± 0.37	29.71 ± 0.41	28.73 ± 0.74	29.40 ± 0.45	29.49 ± 0.58	29.72 ± 0.46	29.82 ± 0.44
LCS and NS per Episode (%)									
No LCS or NS	35.66 ± 0.32	38.65 ± 0.40	30.62 ± 0.33 **	38.84 ± 0.57	30.43 ± 0.74 **	38.42 ± 0.55	30.96 ± 0.52 **	38.69 ± 0.52	30.46 ± 0.42 **
NS only	53.07 ± 0.29	61.35 ± 0.40	39.14 ± 0.32	61.16 ± 0.57	42.37 ± 0.76	61.58 ± 0.55	39.46 ± 0.51	61.31 ± 0.52	37.07 ± 0.46
LCS only	4.85 ± 0.11	--	13.02 ± 0.22	--	11.05 ± 0.51	--	12.91 ± 0.35	--	14.21 ± 0.36
LCS and NS	6.42 ± 0.16	--	17.22 ± 0.27	--	16.15 ± 0.59	--	16.67 ± 0.44	--	18.26 ± 0.38
Time of First Episode (mean ± min)	7:30am ± 2.4	7:39am ± 2.6	7:13am ± 3.0 **	7:37am ± 3.6	7:22am ± 5.4	7:34am ± 3.5	7:04am ± 4.8 **	7:47am ± 3.4	7:16am ± 4.1 **
Time of Last Episode (mean ± min)	8:23pm ± 1.2	8:21pm ± 1.4	8:27pm ± 1.9 *	8:25pm ± 2.6	8:29pm ± 4.0	8:20pm ± 2.2	8:24pm ± 2.9	8:19pm ± 1.9	8:28pm ± 3.0 *
Number of Episodes (n/d, mean)	5.60 ± 0.03	5.42 ± 0.04	5.93 ± 0.03 **	5.56 ± 0.05	6.04 ± 0.08 **	5.44 ± 0.04	5.98 ± 0.06 **	5.26 ± 0.04	5.83 ± 0.04 **
Eating Hours *^5^* (h/d, mean)	12.60 ± 0.04	12.40 ± 0.04	12.97 ± 0.05 **	12.43 ± 0.06	12.86 ± 0.10 **	12.49 ± 0.05	13.09 ± 0.07 **	12.27 ± 0.06	12.93 ± 0.06 **
Eating Hours *^5^* (%)									
Less than 11.0 h/24	20.48 ± 0.51	22.93 ± 0.60	15.96 ± 0.59 **	21.24 ± 0.89	18.28 ± 1.33 *	21.86 ± 0.70	15.32 ± 0.90 **	25.63 ± 0.92	15.21 ± 0.77 **
≥11.0 to <13.0 h/24	28.46 ± 0.41	29.22 ± 0.44	27.06 ± 0.78	30.59 ± 0.88	26.56 ± 1.46	28.09 ± 0.82	24.91 ± 1.23	29.02 ± 0.71	29.02 ± 1.15
≥13.0 to <14.5 h/24	26.03 ± 0.39	25.17 ± 0.43	27.63 ± 0.62	25.63 ± 0.86	26.34 ± 1.37	26.01 ± 0.75	28.89 ± 1.24	23.88 ± 0.76	27.34 ± 0.95
14.5 h or greater/24	25.03 ± 0.54	22.68 ± 0.58	29.35 ± 0.83	22.53 ± 0.82	28.82 ± 1.62	24.03 ± 0.87	30.89 ± 1.29	21.48 ± 0.79	28.43 ± 1.35
Evening/Morning Energy Ratio (median)	1.69 (0.73, 4.00)	1.69 (0.71, 4.30)	1.67 (0.76, 3.59) **	1.84 (0.82, 4.46)	1.79 (0.83, 3.91) **	1.66 (0.70, 4.00)	1.64 (0.76, 3.20) **	1.60 (0.66, 4.48)	1.66 (0.70, 3.87) **
Evening/Morning Energy (%)									
Lower 25th (<0.73, morning)	25.00 ± 0.64	25.51 ± 0.62	24.05 ± 0.74 **	22.81 ± 0.88	22.44 ± 1.47	26.00 ± 0.83	23.19 ± 1.30 **	27.64 ± 1.03	25.60 ± 0.91
25th to 50th (≥0.73 to <1.69)	25.00 ± 0.53	24.35 ± 0.57	26.20 ± 0.77	24.25 ± 0.88	25.39 ± 1.85	24.74 ± 0.88	28.30 ± 1.40	24.05 ± 0.82	24.98 ± 0.98
50th to 75th (≥1.69 to <4.00)	25.00 ± 0.45	23.87 ± 0.50	27.05 ± 0.68	25.63 ± 0.81	27.67 ± 1.38	24.17 ± 0.92	28.87 ± 1.37	21.87 ± 0.68	25.28 ± 1.06
Upper 75th (≥4.00, evening)	25.00 ± 0.39	26.27 ± 0.48	22.70 ± 0.71	27.31 ± 0.89	24.49 ± 1.35	25.08 ± 0.77	19.64 ± 1.16	26.44 ± 0.83	24.13 ± 1.07

*^1^* Eating episode level intake characteristics use the number of eating episodes as the denominator (136,938 total eating episodes reported in NHANES day-1 dietary data). A total of 402,930 items were consumed in 25,411 people (87,270 from 2007 to 2008, 91,460 from 2009 to 2010, 72,466 from 2011 to 2012, 78,729 from 2013 to 2014, and 73,005 from 2015 to 2016). This consisted of 137,023 eating episodes prior to excluding 85 without food or beverage, for a total of 136,938 to be included in the analysis. Up to 26 items were consumed within an eating episode, and persons had up to 18 eating episodes within the 24-h period. *^2^* Person level intake characteristics are shaded and use the applicable NHANES population as the denominator (total population sample *n* = 25,411; projects to 217,802,638 people). *^3^* * = Statistically significant at *p* < 0.05 comparing nutrients by LCS vs. no LCS within BMI categories. ** = Statistically significant at *p* < 0.001 comparing nutrients by LCS vs. no LCS within BMI categories. Abbreviations: US, United States; LCS, low-calorie sweetener; BMI, body mass index; NHANES, National Health and Nutrition Examination Survey; NS, nutritive sweetener.*^4^* Food and beverage additions were included in food only, beverage only, and food and beverage eating episodes. *^5^* Eating hours are the total number of daily hours during which eating episodes were reported.

**Table 2:** Nutrients per eating episode *^1^* and for all eating episodes *^2^* (i.e., at the person level) across the day in US adults aged ≥19 y by LCS intake and BMI category (NHANES 2007–2016). *^3^*.

	Total Population	Total Population		Normal Weight (29.4%)		Overweight (33.6%)		Obese (37.1%)	
		No LCS (64.8%)	LCS (35.2%)	No LCS (21.2%)	LCS (8.2%)	No LCS (21.7%)	LCS (11.9%)	No LCS (21.9%)	LCS (15.1%)
Nutrients per episode (mean ± SEM)									
Grams (g)	630 ± 5	644 ± 5	605 ± 6 **	616 ± 8	568 ± 11 **	648 ± 8	603 ± 9 **	668 ± 7	628 ± 7 **
Energy (kcal)	383 ± 2	406 ± 3	344 ± 3 **	400 ± 5	327 ± 5 **	408 ± 4	347 ± 5 **	408 ± 4	352 ± 4 **
Energy from Food (kcal)	292 ± 2	303 ± 2	274 ± 2 **	298 ± 4	255 ± 4 **	304 ± 3	273 ± 4 **	306 ± 3	286 ± 4 **
Energy from Beverage (kcal)	66 ± 1	79 ± 1	46 ± 1 **	78 ± 2	46 ± 2 **	79 ± 2	49 ± 2 **	79 ± 1	43 ± 1 **
Energy from FBA (kcal)	24 ± 0.3	24 ± 0.4	25 ± 0.5	24 ± 1	27 ± 1	25 ± 1	25 ± 1	23 ± 1	23 ± 1
Energy density (kcal/g)	1.14 ± 0.01	1.17 ± 0.01	1.09 ± 0.01 **	1.19 ± 0.01	1.13 ± 0.02 *	1.15 ± 0.01	1.07 ± 0.02 **	1.16 ± 0.01	1.08 ± 0.02 **
Protein (g)	14.8 ± 0.10	15.3 ± 0.12	14.1 ± 0.13 **	15.0 ± 0.21	13.2 ± 0.3 **	15.6 ± 0.2	14.3 ± 0.2 **	15.2 ± 0.2	14.5 ± 0.2 **
Carbohydrate (g)	45.7 ± 0.27	49.3 ± 0.35	39.6 ± 0.31 **	48.9 ± 0.62	39.2 ± 0.7 **	49.2 ± 0.5	39.5 ± 0.6 **	49.8 ± 0.8	39.9 ± 0.5 **
Total Sugars (g)	20.3 ± 0.18	22.6 ± 0.24	16.3 ± 0.16 **	22.1 ± 0.38	16.6 ± 0.3 **	22.7 ± 0.5	16.2 ± 0.3 **	23.1 ± 0.3	16.1 ± 0.3 **
Dietary Fiber (g)	3.05 ± 0.03	3.09 ± 0.02	2.97 ± 0.04*	3.15 ± 0.05	3.10 ± 0.08	3.12 ± 0.04	3.01 ± 0.06	3.01 ± 0.04	2.87 ± 0.04 *
Total Fat (g)	14.7 ± 0.10	15.2 ± 0.12	13.7 ± 0.14 **	14.7 ± 0.21	12.5 ± 0.3 **	15.4 ± 0.2	13.7 ± 0.2 **	15.6 ± 0.2	14.5 ± 0.2 **
Nutrients for all eating episodes (Person level *^2^*; mean ± SEM)									
Energy (kcal)	2143 ± 9	2197 ± 13	2044 ± 14 **	2225 ± 24	1977 ± 26 **	2220 ± 19	2075 ± 26 **	2147 ± 19	2055 ± 20 *
Protein (g)	83.1 ± 0.5	82.7 ± 0.5	83.8 ± 0.7	83.2 ± 1.0	79.7 ± 1.0 *	84.7 ± 1.1	85.4 ± 1.1	80.1 ± 0.7	84.7 ± 1.0 **
Carbohydrate (g)	256 ± 1	267 ± 2	235 ± 2 **	272 ± 3	237.1 ± 4 **	267 ± 2	236 ± 3 **	262 ± 3	232 ± 2 **
Total Sugars (g)	113 ± 1	123 ± 1	96.5 ± 0.9 **	123 ± 2	100.2 ± 2 **	123 ± 1.	96.9 ± 1.7 **	122 ± 2	94 ± 1.3 **
Dietary Fiber (g)	17.1 ± 0.2	16.8 ± 0.2	17.6 ± 0.2 **	17.6 ± 0.3	18.7 ± 0.4 *	17.0 ± 0.3	18.0 ± 0.4 *	15.8 ± 0.2	16.8 ± 0.2 *
Total Fat (g)	82.1 ± 0.5	82.5 ± 0.6	81.5 ± 0.7	81.9 ± 1.0	75.7 ± 1.4 **	83.6 ± 0.9	81.9 ± 1.3	81.8 ± 0.9	84.4 ± 1.2

*^1^* Eating episode level intake characteristics use the number of eating episodes as the denominator (136,938 total eating episodes reported in NHANES day-1 dietary data). A total of 402,930 items were consumed in 25,411 people (87,270 from 2007 to 2008, 91,460 from 2009 to 2010, 72,466 from 2011 to 2012, 78,729 from 2013 to 2014, and 73,005 from 2015 to 2016). This consisted of 137,023 eating episodes prior to excluding 85 without food or beverage, for a total of 136,938 to be included in the analysis. Up to 26 items were consumed within an eating episode, and persons had up to 18 eating episodes within the 24-h period. *^2^* Person level intake characteristics use the applicable NHANES population as the denominator (total population sample *n* = 25,411; projects to 217,802,638 people). ^3 *^ = Statistically significant at *p* < 0.05 comparing nutrients by LCS vs. no LCS item consumption within BMI categories. ** = Statistically significant at *p* < 0.001 comparing nutrients by LCS vs. no LCS item consumption within BMI categories. Abbreviations: US, United States; LCS, low-calorie sweetener; BMI, body mass index; NHANES, National Health and Nutrition Examination Survey; SEM; standard error of the mean; FBA, food and beverage addition.

**Table 3:** Estimated mean BMI differences per indicated unit of each variable after adjustment as specified for each model. Separate models were run for each variable (NHANES 2007–2016).

	Mean BMI Difference per Specified Unit (95% CI)		
Variable	Initial Models *^1^*	Secondary Models *^2^*	Final Models *^3^*
LCS (yes versus no)	1.61 (1.32, 1.91)	----	----
Number of Episodes (n/day)	−0.16 (−0.23, −0.08)	−0.20 (−0.27, −0.12)	−0.22 (−0.29, −0.14)
Eating Hours *^4^* (h/day)	−0.02 (−0.07, 0.02)	−0.05 (−0.09, 0.004)	−0.05 (−0.10, −0.001)
Eating Hours *^4^* (categories)			
Less than 11.0 h/24	Referent	Referent	Referent
≥11.0 to <13.0 h/24	−0.21 (−0.55, 0.13)	−0.25 (−0.61, 0.10)	−0.27 (−0.62, 0.09)
≥13.0 to <14.5 h/24	−0.32 (−0.64, −0.001)	−0.42 (−0.74, −0.10)	−0.44 (−0.76, −0.12)
14.5 h or greater/24	−0.32 (−0.69, 0.05)	−0.47 (−0.84, −0.11)	−0.50 (−0.88, −0.13)
Evening/Morning Energy			
Lower 25th (<0.73, morning)	Referent	Referent	Referent
25th to 50th (≥0.73 to <1.69)	−0.06 (−0.52, 0.08)	−0.24 (−0.54, 0.05)	−0.25 (−0.55, 0.05)
50th to 75th (≥1.69 to <4.00)	−0.36 (−0.68, −0.05)	−0.41 (−0.73, −0.09)	−0.42 (−0.74, −0.10)
Upper 75th (≥4.00, evening)	−0.06 (−0.41, 0.29)	−0.06 (−0.41, 0.29)	−0.06 (−0.41, 0.28)

*^1^* Initial models are adjusted for age category, gender, race–ethnic group, and education level. *^2^* Secondary models are additionally adjusted for LCS items (yes versus no). *^3^* Final models are additionally adjusted for LCS items as well as total energy consumption. Abbreviations: BMI, body mass index; NHANES, National Health and Nutrition Examination Survey; LCS, low-calorie sweetener; CI, confidence interval. *^4^* Eating hours are the total number of daily hours during which eating episodes were reported.

**Table 4:** Estimated mean BMI differences per indicated unit of each variable in consumers and non-consumers of LCS (NHANES 2007–2016). ^1^.

	BMI—Difference per Specified Unit (Beta Values with 95% CI)		
	Stratified by LCS		
Variable	LCS—No	LCS—Yes	*p*-Value Interaction
Number of Episodes (n/d)	−0.24 (−0.32, −0.15)	−0.18 (−0.31, −0.06)	0.4407
Eating Hours ^2^ (h/d)	−0.06 (−0.12, −0.01)	−0.02 (−0.12, 0.09)	0.4231
Eating Hours ^2^ (categories)			
Less than 11.0 h/24	Referent	Referent	
≥11.0 to <13.0 h/24	−0.60 (−0.99, −0.22)	0.50 (−0.16, 1.16)	0.0035
≥13.0 to <14.5 h/24	−0.56 (−0.93, −0.19)	−0.07 (−0.70, 0.56)	0.1823
14.5 h or greater/24	−0.69 (−1.08, −0.30)	−0.04 (−0.76, 0.68)	0.1027
Evening/Morning Energy			
Lower 25th (<0.73, morning)	Referent	Referent	
25th to 50th (≥0.73 to <1.69)	−0.30 (−0.63, 0.04)	−0.16 (−0.73, 0.42)	0.6697
50th to 75th (≥1.69 to <4.00)	−0.47 (−0.82, −0.12)	−0.33 (−0.91, 0.25)	0.6658
Upper 75th (≥4.00, evening)	−0.13 (−0.52, 0.26)	0.06 (−0.52, 0.65)	0.5656

^1^ Models are adjusted for age category, gender, race–ethnic group, education level, and total energy consumption. Abbreviations: BMI, body mass index; LCS, low-calorie sweetener; NHANES, National Health and Nutrition Examination Survey; CI, confidence interval. ^2^ Eating hours are the total number of daily hours during which eating episodes were reported.

## Supplementary Materials

The following is available online at https://www.mdpi.com/2072-6643/12/9/2566/s1, Figure S1: Flow chart defining study population and exclusions at person level. Table S1: Characteristics of the US adult population NHANES (2007-2016).

## Abbreviations

BMI	body mass index
CI	confidence interval
CSFII	Continuing Survey of Food Intakes of Individuals
DG	Dietary Guidelines for Americans
DGAC	Dietary Guidelines Advisory Committee
FBA	food and beverage additions
FDA	United States Food and Drug Administration
INTERMAP	International Study on Macro/Micronutrients and Blood Pressure
LCS	low-calorie sweetener
NCHS	National Center for Health Statistics
NHANES	National Health and Nutrition Examination Survey
NHW	non-Hispanic white
NHB	non-Hispanic black
NS	nutritive sweeteners
US	United States
USDA	United States Department of Agriculture
y	year
